# Gaps in Iranian School-leavers’ Current Knowledge of Nutrition and Food Systems

**Published:** 2017-11

**Authors:** Sanaz SADEGHOLVAD, Heather YEATMAN, Nasrin OMIDVAR, Anne-Maree PARRISH, Anthony WORSLEY

**Affiliations:** 1. Dept. of Health and Society, Faculty of Social Sciences, University of Wollongong, Wollongong, Australia; 2. Dept. of Community Nutrition, Faculty of Nutrition Sciences and Food Technology, Shahid Beheshti University of Medical Sciences, Tehran, Iran; 3. Center for Physical Activity Research, School of Exercise and Nutrition Sciences, Deakin University, Burwood, Melbourne, Australia

## Dear Editor-in-Chief

There is a scarcity of literature exploring Iranian people’s knowledge of a wide range of nutrition and food system (N&FS) issues. Twenty-six experienced Iranian food-related experts from different provinces of Iran (Fars, Tehran, Isfahan and Gilan) were interviewed between September 2012 and December 2012 to explore their views of gaps in Iranian school-leavers’ current knowledge of N&FS. Thematic analysis approach was employed to analyze the qualitative data. Five key themes emerged from interview transcripts, briefly described here:

1. Generally poor, and scattered knowledge across most of the N&FS areas; even those related to fundamental nutrition issues such as appropriate nutrition for growth and development; malnutrition; food and chronic diseases; foodborne illnesses and other food safety issues reported by most of the participants. Previous Iranian literature has not directly assessed students’ and school-leavers’ knowledge of identified fundamental nutrition issues.
2. Substantial lack of specific knowledge of food systems; in relation to agriculture, food production, food processing and environmental issues associated with food systems. The major reason for this knowledge deficit was identified as a lack of attention to food systems topics within schools’ education curricula and general societal education (e.g. through mass media). There is absence of literature exploring Iranian populations’ knowledge of food systems issues. However, the importance of food systems knowledge has been reported in international literature as “our food choices not only affect our health, but they can also have wide-ranging implications for the sustainability of food production and natural resources” (1)
3. Knowledge and attractiveness of N&FS topics to students; school-leavers have some knowledge about topics that are appealing to them, particularly those related to their physical appearance (e.g. weight issues), and physical activity and sports performance. However, experts expressed their concerns about the accuracy of students’ and school-leavers’ knowledge of these issues, due to the unreliability of their information sources, for example, gymnasiums. International literature also has raised concerns about poor quality of some information provided to young population groups. A Brazilian study of the nutrition knowledge and dietary recommendations by coaches to adolescent athletes found that coaches recommended dietary practices such as very lowfat diets; over emphasize on proteins, and perpetuated food myths (2).
4. Determinants of current N&FS knowledge, that positively or negatively affected the current N&FS knowledge of Iranian school-leavers. These included the content of school textbooks; the attractiveness of food and nutrition topics for students; students’ geographical location (urban or rural); the family environment (parent’s occupation, for example farmer or office worker, and parents’ own knowledge and practices of nutrition and food systems); mass media activities; and duration of food and nutrition education programs provided for students. Overall, the participants believed that at present Iranian schools and mass media do not use their potentials to deliver important nutrition and food systems information to Iranian population groups. The participants’ identification of insufficient nutrition information in schoolbooks is consistent with a recent Iranian study that concluded the potential of primary school textbooks to deliver health-related messages (e.g. nutrition) has not been fully utilized (3). These findings differ from the common determinants of nutrition knowledge reported in existing worldwide literature, which includes educational level, age of students and family occupation (4, 5). Such differences may have arisen because most of the previous studies focused on nutrition knowledge alone, whereas in the present study knowledge of nutrition issues were explored in conjunction with knowledge of food systems. Additionally, previous studies did not explore experts’ views on this matter. Experts are likely to take a wider perspective of influences on knowledge acquisition than the limited demographic indicators usually reported in quantitative surveys of knowledge.
5. Key barriers to students’ study of N&FS at the school level, including food and nutrition information covered in school textbooks is inadequate and mostly not useful for everyday living; and some major areas of study taken by students at high schools are limiting. Students studying in the science stream receive some food-related lessons; however, those studying in social sciences, mathematics and other majors are likely to be deprived of nutrition and food systems education.

The results of this study are important as they can inform the identification of neglected components of N&FS education within school curricula. More broadly, the findings can assist food and nutrition educators, curriculum developers and related policy makers to improve the current N&FS education programs in schools in Iran. Knowledge is power and better informed school-leavers are more likely to make healthy and sustaining food choices.
